# SPP1-associated pathomics features for prognostic modeling in pancreatic ductal adenocarcinoma

**DOI:** 10.3389/fonc.2026.1923860

**Published:** 2026-10-23

**Authors:** Jinming Zhao, Mingjun Li, Jialiang Xing, Yunsong Chen, Xingguo Song

**Affiliations:** 1 Department of Clinical Laboratory, Shandong Cancer Hospital and Institute, Shandong First Medical University and Shandong Academy of Medical Sciences, Jinan, Shandong, China; 2 Department of Research and Education, Jinan Center for Disease Control and Prevention, Jinan, Shandong, China; 3 General Surgery, The 5th People’s Hospital of Jinan, Jinan, Shandong, China; 4 Shandong Provincial Key Laboratory of Precision Oncology, Shandong Cancer Hospital and Institute, Shandong First Medical University and Shandong Academy of Medical Sciences, Jinan, Shandong, China

**Keywords:** secreted phosphoprotein 1 (SPP1), pancreatic ductal adenocarcinoma (PDAC), pathomics, prognostic model, tumor microenvironment (TME), immune infiltration

## Abstract

**Background:**

Pancreatic ductal adenocarcinoma (PDAC) is a highly lethal malignancy characterized by an immunosuppressive tumor microenvironment (TME).

**Methods:**

In this study, we investigated the prognostic and immunological relevance of SPP1 in PDAC through integrative analyses of public transcriptomic datasets, single-cell resources, and tissue-based assessments. Furthermore, leveraging high-throughput pathomics via whole-slide imaging and advanced computational analytics, we developed and internally validated a prognostic model incorporating two pathomics features (Zernike_2_2, Hematoxylin_4of4) together with age.

**Results:**

SPP1 was found to be upregulated in PDAC overall compared to normal tissue, whereas its expression was lower in TLS-enriched regions relative to adjacent normal tissues. Multidimensional analysis revealed correlations between SPP1 expression and macrophage infiltration estimates. Moreover, low SPP1 expression was associated with favorable prognosis in PDAC patients. The pathomics-based prognostic model demonstrated good discriminative performance (bootstrap-corrected C-index = 0.715; corrected 12-month AUC = 0.766; apparent AUC = 0.866) and significant survival stratification between high- and low-risk groups (log-rank P < 0.0001).

**Conclusions:**

These findings suggest that SPP1-associated histoarchitectural patterns captured by pathomics may provide prognostic information, and that SPP1 represents a potential immunomodulatory factor and a candidate prognostic biomarker for PDAC.

## Introduction

Pancreatic ductal adenocarcinoma (PDAC) remains one of the most lethal malignancies worldwide (1). Despite advances in surgical techniques and systemic therapies, the 5-year survival rate remains below 12%, with distant metastasis carrying a particularly dismal prognosis of less than 5% (2). This poor outcome is largely attributed to late diagnosis, aggressive metastatic behavior, and an extraordinarily complex and immunosuppressive tumor microenvironment (TME) (3). Compounding this challenge, the remarkable interpatient heterogeneity of PDAC further limits the efficacy of current chemo- and immunotherapies, underscoring the pressing need for reliable biomarkers and tools for prognostic stratification to guide individualized treatment decisions (3).

Secreted phosphoprotein 1 (SPP1, also known as osteopontin) is a multifunctional secreted glycoprotein that has emerged as a candidate prognostic biomarker in multiple cancers (4). In PDAC, SPP1 is upregulated in tumor tissues compared to normal pancreas and has been shown to correlate with tumor progression and adverse clinical outcomes (5). Recent studies have demonstrated that SPP1 expression is subject to multilayered regulation, including genetic variants, epigenetic modifications, and long non-coding RNAs (6), highlighting the complexity of its functional roles in disease contexts. Functionally, SPP1 has been implicated in both tumor-intrinsic processes, such as mesenchymal cell fate determination and epithelial-mesenchymal transition (7, 8), and tumor-extrinsic immune modulation. In particular, SPP1^+^ tumor-associated macrophages have emerged as key drivers of immunosuppression and therapeutic resistance in PDAC: they interact with PRRX2^+^ epithelial cells through the TGFB1/TGFBR2 axis to promote gemcitabine resistance (9), and their CXCL9-low/SPP1-high phenotype is associated with a non-inflamed TME that can be reversed by myeloid-targeting therapeutic strategies (10). Moreover, the presence of tertiary lymphoid structures (TLSs) within the PDAC TME has been associated with improved prognosis and enhanced anti-tumor immune responses (11), and SPP1 has been identified as a TLS-associated gene with prognostic significance in PDAC (12, 13). Collectively, these findings implicate SPP1 as a key link between macrophage-driven immune suppression, TLS-associated immune modulation, and patient outcomes in PDAC. To bridge these observations with prognostic modeling, we characterized SPP1 expression in relation to macrophage infiltration and developed a pathomics-based prognostic model incorporating SPP1-associated histoarchitectural features.

In recent years, computational pathology and pathomics have emerged as powerful tools for extracting quantitative morphological features from routine whole-slide images, enabling objective and reproducible assessment of tumor histoarchitecture (14–16). Machine learning-based pathomics models have shown promise in predicting overall survival (OS) and immune status in PDAC patients (17). For instance, a deep learning-based pathomics score derived from H&E-stained whole slide images across 864 PDAC patients from two centers served as an independent prognostic factor and surpassed clinical models in predicting 1-, 2-, and 3-year survival (14). Similarly, unsupervised machine learning applied to pathological features from TCGA-PAAD data identified feature clusters significantly associated with poor prognosis (18). More recently, a histopathomics-based survival prediction system (HPSurv) integrating tumor heterogeneity quantification and a survival Transformer achieved a mean C-index of 0.761 (19). However, existing approaches predominantly rely on black-box deep learning architectures, which often lack interpretability and provide limited insight into the specific histoarchitectural features driving prognostic predictions (20). Moreover, integrative models combining pathomics with radiomics and clinical factors have demonstrated improved predictive performance, underscoring the complementary value of multi-scale features (21). Nevertheless, the link between molecular signatures such as SPP1 and quantifiable pathomics features remains largely unexplored, and few studies have operationalized this connection within an interpretable prognostic framework.

Prediction models are increasingly used to support clinical decision-making, but their translation depends on transparent reporting and rigorous validation. The TRIPOD statement and PROBAST provide complementary guidance for reporting and appraising multivariable prediction models (22, 23). In parallel, computational pathology and digital pathology-based analytics enable objective quantification of tumor histomorphology and microenvironment organization (24). However, many pathomics models remain constrained by insufficient dataset standardization, lack of multicenter validation, and limited interpretability. These methodological considerations are particularly relevant to PDAC, where molecular markers such as SPP1 may be linked to histoarchitectural and immune features.

In this study, we aimed to bridge this gap by leveraging public transcriptomic and proteomic datasets, single-cell resources, and tissue-based assessments to comprehensively characterize SPP1 expression and its immunological associations in PDAC. More importantly, we developed and internally validated an interpretable prognostic model by integrating SPP1-associated pathomics features with clinical covariates. Using a multi-algorithm consensus strategy, we constructed a three-feature Cox model, which demonstrated good discriminative performance and significant survival stratification. Our findings suggest that SPP1-associated histoarchitectural patterns captured by pathomics may offer prognostic value, positioning SPP1 as a potential immunomodulatory factor and candidate prognostic biomarker in PDAC.

## Materials and methods

### Patients and tissue specimens

This study utilized a commercially available tissue microarray (HPanA120Su02, Shanghai Outdo Biotech Co., Ltd.). We included patients with pathologically confirmed PDAC who underwent curative-intent resection between January 2009 and August 2013 and had complete clinical follow-up data. Patients with a history of other malignancies or incomplete follow-up records were excluded. Follow-up was censored in November 2014, with a duration ranging from 1.2 to 5.8 years. Clinical staging was according to the 7th edition of the AJCC staging system. Among the initial specimens, two were excluded from pathomics analysis due to poor slide quality (severe tissue folding or uneven staining) that prevented stable feature extraction, leaving 63 evaluable cases. Overall survival was defined as the time from surgery to death or last follow-up; patients alive or lost to follow-up were censored.

The tissue microarray was approved for research use by the company’s Ethics Committee (Approval No. SHYJS-CP-2001007), and all patients provided written informed consent for sample collection. All methods were performed in accordance with relevant guidelines and regulations and in compliance with the Declaration of Helsinki.

### Bioinformatics analysis

SPP1 expression was analyzed across pan-cancer datasets from The Cancer Genome Atlas (TCGA; https://portal.gdc.cancer.gov/) and the Genotype-Tissue Expression (GTEx) project (https://gtexportal.org/home/). Six datasets were retrieved from the NCBI GEO database (http://www.ncbi.nlm.nih.gov/geo): GSE15471 (39 PDAC tumors/39 normals), GSE62165 (118 PDAC tumors/13 normals), GSE28735 (45 PDAC tumors/45 normals), GSE62452 (69 PDAC tumors/61 normals), GSE226840 (14 PDAC-associated tertiary TLSs/5 adjacent normals), and GSE85916 (80 tumors). The cohorts GSE15471, GSE62165, GSE28735, and GSE62452 were used for SPP1 differential expression analysis; GSE226840 facilitated differentially expressed gene (DEG) identification; and GSE85916 functioned as the validation cohort for target genes (25). DEGs were screened using filter criteria of adjusted P < 0.05 and |logFC| > 1. Volcano plots and heatmaps were constructed using OmicShare (26).

The Kaplan-Meier (KM) curve was employed to evaluate the prognostic significance of SPP1 in PDAC, demonstrating significant differences in both OS and recurrence-free survival (RFS) (log-rank P < 0.05) (27). Pan-cancer expression profiles of SPP1 across tumor and normal tissues were analyzed using the Sangerbox platform (28). Protein expression of SPP1 was validated using the CPTAC dataset accessed via the Human Protein Atlas (HPA) portal (29). Single-cell transcriptomic profiling was performed using scCancerExplorer (30) and TISCH2. Immune infiltration landscapes were quantified via TIMER 2.0 platforms: the Immune-Gene module specifically mapped macrophage subtype correlations and the Immune-Outcome module established clinical prognostic associations in pancreatic adenocarcinoma (PAAD) (31).

### Immunohistochemistry and image quantification

Tissue microarray sections were deparaffinized with an environmentally friendly dewaxing solution (G1128) and rehydrated through a graded ethanol series. Antigen retrieval was performed using an antigen retrieval apparatus. The sections were then washed with PBS (pH 7.4) three times for 3 min each at room temperature. Endogenous peroxidase activity was blocked by incubation with 3% H_2_O_2_ for 15 min, followed by three PBS washes. Non-specific binding was blocked with normal goat serum (G1208, diluted 1∶9 in PBS) for 30 min at room temperature. After blocking, the sections were incubated with a primary antibody against SPP1 (Servicebio, Wuhan, China; GB11500; diluted 1∶1000 in PBS) overnight at 4 °C in a humidified chamber. Following three washes with PBS, the sections were incubated with HRP-conjugated goat anti-rabbit IgG (Servicebio, GB23303; diluted 1∶200 in PBS) for 1 h at room temperature. After additional PBS washes, color development was achieved using freshly prepared DAB substrate (Servicebio, G1212-200T; diluted 1∶100), and the reaction was monitored under a microscope; it was terminated by rinsing with tap water when a brownish-yellow positive signal appeared with minimal background staining. The sections were then counterstained with hematoxylin, differentiated in the differentiation solution for a few seconds, blued in bluing solution for approximately 20 s, and washed under running water. Finally, the sections were dehydrated through graded ethanol, cleared in xylene, and mounted with neutral balsam.

Whole-slide images were acquired using a digital scanner and analyzed with the Aipathwell platform (Servicebio, China). The positive area ratio was calculated as (SPP1-positive area/total tissue area) × 100%. Positive signals were identified by color deconvolution-based automated thresholding of DAB staining (brown), and the thresholds were verified by two independent observers to ensure consistency across all sections.

### Extraction of quantitative image features

An image processing pipeline was developed using CellProfiler (version 4.2.8, Broad Institute of MIT and Harvard, Cambridge, MA, USA) modules for segmentation and feature extraction. Color deconvolution was performed using the ‘UnmixColors’ module to separate hematoxylin and DAB staining channels. Automated segmentation of nuclei and cytoplasm was performed using “IdentifyPrimaryObjects” and “IdentifySecondaryObjects” modules. Quantitative features, including object shape, size, texture, and pixel intensity distribution, were extracted using the “MeasureObjectSizeShape”, “MeasureTexture”, “MeasureObjectIntensity” and “MeasureObjectIntensityDistribution” modules. In total, 361 handcrafted quantitative pathomics features were extracted from each tissue core for downstream analysis.

### Model building strategy and evaluation

To construct the prognostic model, we employed an ensemble feature selection strategy combining LASSO, random forest, and decision tree. LASSO regression was applied to the 361 pathomics features with 10-fold cross-validation (200 λ values, seed = 30) to select candidate features. These candidate features were then combined with three clinical variables (sex, age, and tumor volume), forming a variable pool. Random forest and decision tree, both with shallow trees (max_depth = 3) to prevent overfitting, were subsequently applied to this pool; random seeds were set to ensure reproducibility. A Cox proportional-hazards regression model was then constructed using the retained features.

Model performance was evaluated using KM survival curves with log-rank tests, time-dependent ROC curves, calibration curves, and decision curve analysis (DCA). SHAP (SHapley Additive exPlanations) analysis was performed to evaluate the contribution of each feature and to facilitate model interpretability, providing insights into the direction and magnitude of feature effects on prognostic predictions. Internal validation was performed using 1,000 bootstrap resampling iterations to obtain optimism-corrected performance estimates, with 95% bootstrap confidence intervals (CI) calculated for the C-index. The proportional-hazards assumption was tested using Schoenfeld residual tests for all variables in the final model, and the results were visually confirmed by plotting scaled Schoenfeld residuals against time.

### Statistical analysis

All statistical analyses were performed using R software (version 4.4.2, R Foundation for Statistical Computing, Vienna, Austria) and GraphPad Prism (version 9.5.0, GraphPad Software Inc., San Diego, CA, USA). Differences in SPP1 expression between groups were compared using the Wilcoxon rank-sum test. Survival analysis was performed using KM curves with log-rank tests and Cox proportional-hazards regression models to estimate hazard ratios (HR) with 95% CI. LASSO regression, random forest, and decision tree were implemented in Python 3.10 using the MyLab + i-Research platform (version 4.9.6, Roche Diagnostics, Shanghai, China) for feature selection. All tests were two-sided, and a P value < 0.05 was considered statistically significant.

## Results

### SPP1 is downregulated in TLS-enriched regions but upregulated in PDAC

Recent studies have demonstrated that the presence of TLS in PDAC correlates with increased immune cell infiltration and improved clinical outcomes in TLS-positive (TLS+) patients (11, 32). To identify potential prognostic biomarkers, we performed differential gene expression analysis between TLS-enriched regions (n=14) and adjacent normal tissues (n=5) from the GSE226840 dataset, applying stringent statistical thresholds (|log_2_ fold change|>1; adjusted p-value<0.05). This analysis revealed 85 DEGs, comprising 52 upregulated and 33 downregulated genes, with SPP1 identified among the downregulated genes in TLS-enriched regions (**Figures 1A, B**).

**Figure 1:**
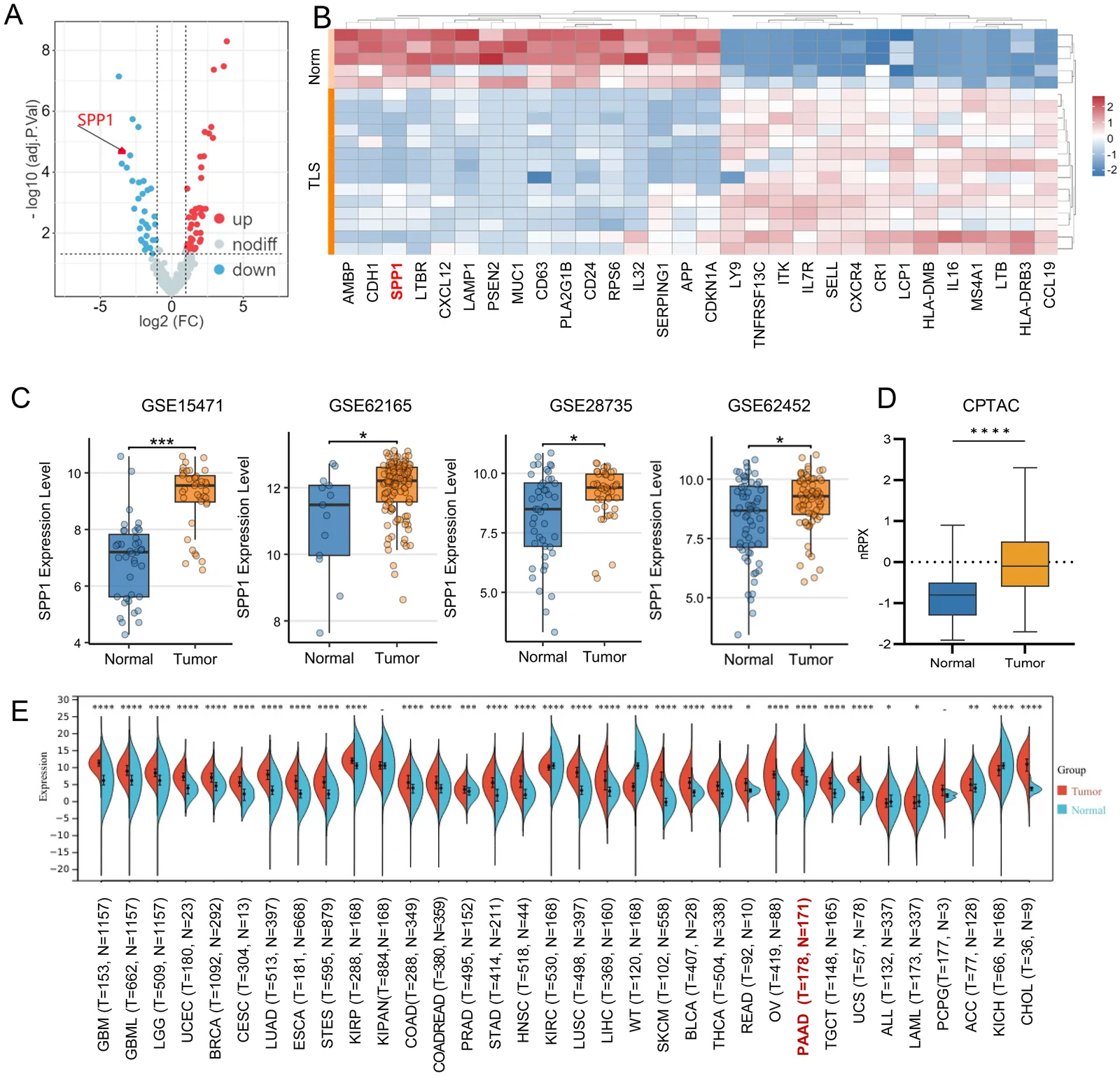
SPP1 is downregulated in TLS-enriched regions but upregulated in PDAC. **(A)** Volcano plot of DEGs between TLS-enriched regions (n = 14) and adjacent normal tissues (n = 5) from the GSE226840 dataset. Red and blue dots represent significantly upregulated and downregulated genes, respectively. **(B)** Heatmap showing the expression profiles of 85 DEGs between TLS-enriched regions and adjacent normal tissues. **(C)** SPP1 mRNA expression in PDAC tumors compared with normal tissues across four independent GEO cohorts (GSE15471, GSE62165, GSE28735, GSE62452). **(D)** SPP1 protein expression in PDAC tumors and normal tissues from the CPTAC dataset. **(E)** Pan-cancer expression of SPP1 between tumor and normal tissues across multiple cancer types, based on TCGA and GTEx datasets via the Sangerbox platform. *p < 0.05, ***P < 0.001, ****P < 0.0001. PDAC, pancreatic ductal adenocarcinoma; DEGs, differentially expressed genes.

Systematic validation across four independent GEO cohorts (GSE15471, GSE62165, GSE28735, GSE62452) confirmed consistent tumor-specific elevation of SPP1 mRNA levels (Wilcoxon rank-sum test, p< 0.05) (**Figure 1C**), a pattern further corroborated at the protein level using the CPTAC dataset (**Figure 1D**, p < 0.001). We further investigated SPP1 expression patterns across various cancers. As shown in **Figure 1E**, comprehensive pan-cancer analysis integrating TCGA and GTEx datasets (processed via the Sangerbox platform) revealed significant SPP1 overexpression across multiple solid tumors, with PAAD showing particularly pronounced transcriptional upregulation.

### SPP1 expression correlates with prognosis in PDAC patients

We next evaluated the prognostic significance of SPP1 expression across independent cohorts systematically. In the TCGA-PAAD cohort, patients with low SPP1 expression demonstrated significantly better OS (HR = 2.65, 95% CI: 1.49-4.71, log-rank P = 0.00059) and RFS (HR = 5.13, 95% CI: 1.70–15.46, log-rank P = 0.0014) compared with those with high expression (**Figure 2A**). These findings were validated in the independent GSE85916 cohort, where low SPP1 expression was also associated with favorable prognosis (HR = 1.97, 95% CI: 1.16–3.36, P = 0.0124) (**Figure 2B**).

**Figure 2:**
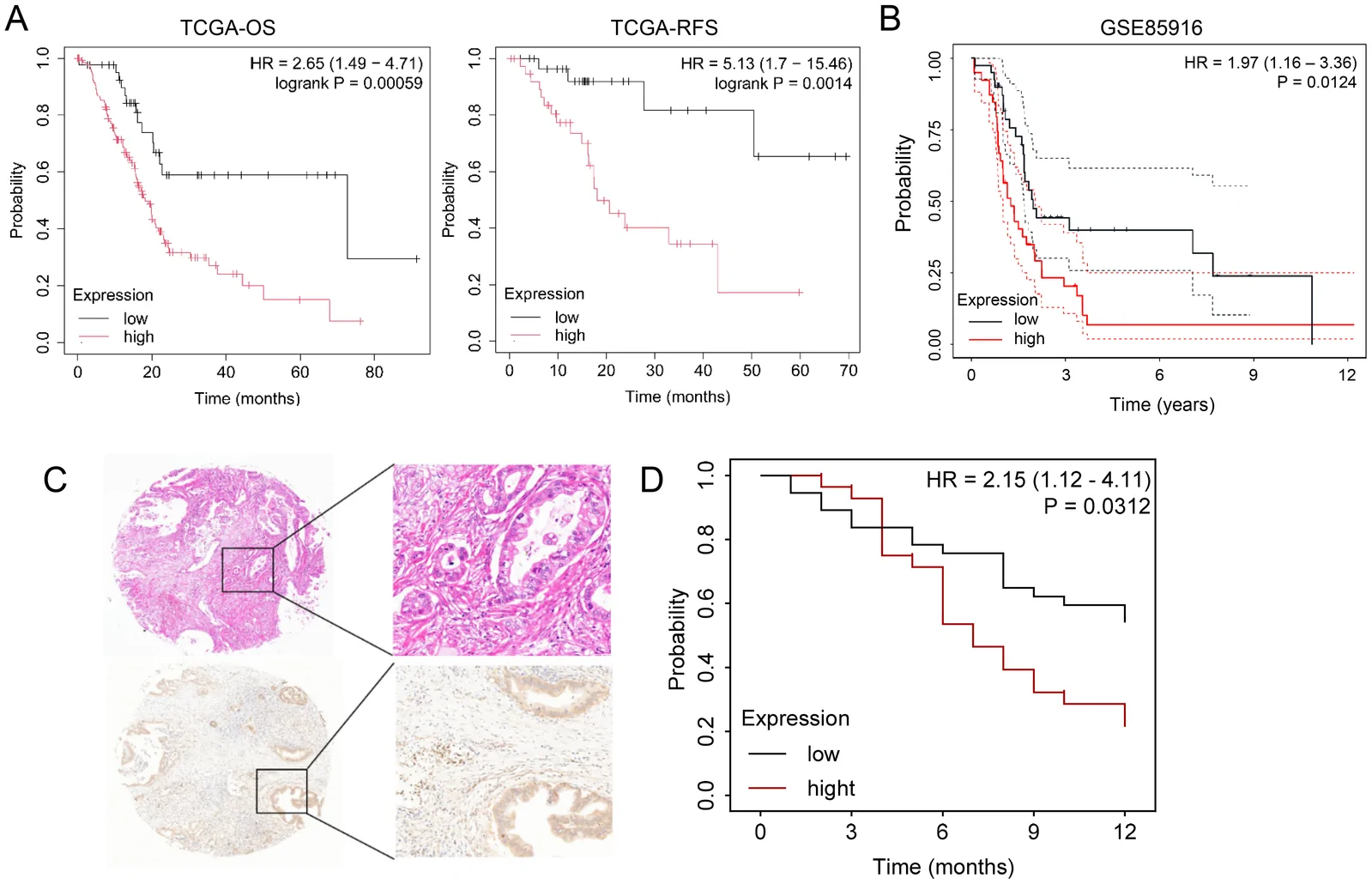
SPP1 expression correlates with prognosis in PDAC patients. **(A)** KM curves for OS and RFS in TCGA-PAAD patients stratified by median SPP1 expression. **(B)** KM curve for overall survival in the independent GSE85916 validation cohort stratified by median SPP1 expression. **(C)** H&E staining and IHC analysis of PDAC tissues. **(D)** KM curve for overall survival in the IHC tissue cohort (n = 63) stratified by SPP1 positive area ratio. KM, Kaplan-Meier; PAAD, pancreatic adenocarcinoma; HR, hazard ratio; CI, confidence interval; OS, overall survival; RFS, recurrence-free survival.

To determine whether SPP1 protein expression similarly impacts patient outcomes, we quantified SPP1 expression in 63 evaluable PDAC specimens through whole-slide digital pathology with representative H&E and IHC staining shown in **Figure 2C**. Based on the positive area ratio, patients were stratified into low- and high-expression groups using the mean value as the cutoff. KM analysis revealed that patients with low SPP1 protein expression had significantly better OS than those with high expression (HR = 2.15, 95% CI: 1.12–4.11, P = 0.0312) (**Figure 2D**). These results support that SPP1 expression, at both the transcriptomic and protein levels, is significantly associated with prognosis in PDAC.

### SPP1-associated pathomics feature selection for prognostic modeling

To identify SPP1-associated histoarchitectural features for prognostic modeling, we performed pathomics analysis on 63 evaluable PDAC tissue sections (two specimens were excluded due to poor slide quality). A total of 361 handcrafted quantitative features, covering object shape, size, texture, and pixel intensity distribution, were extracted from each tissue core using CellProfiler (**Figure 3A**).

**Figure 3:**
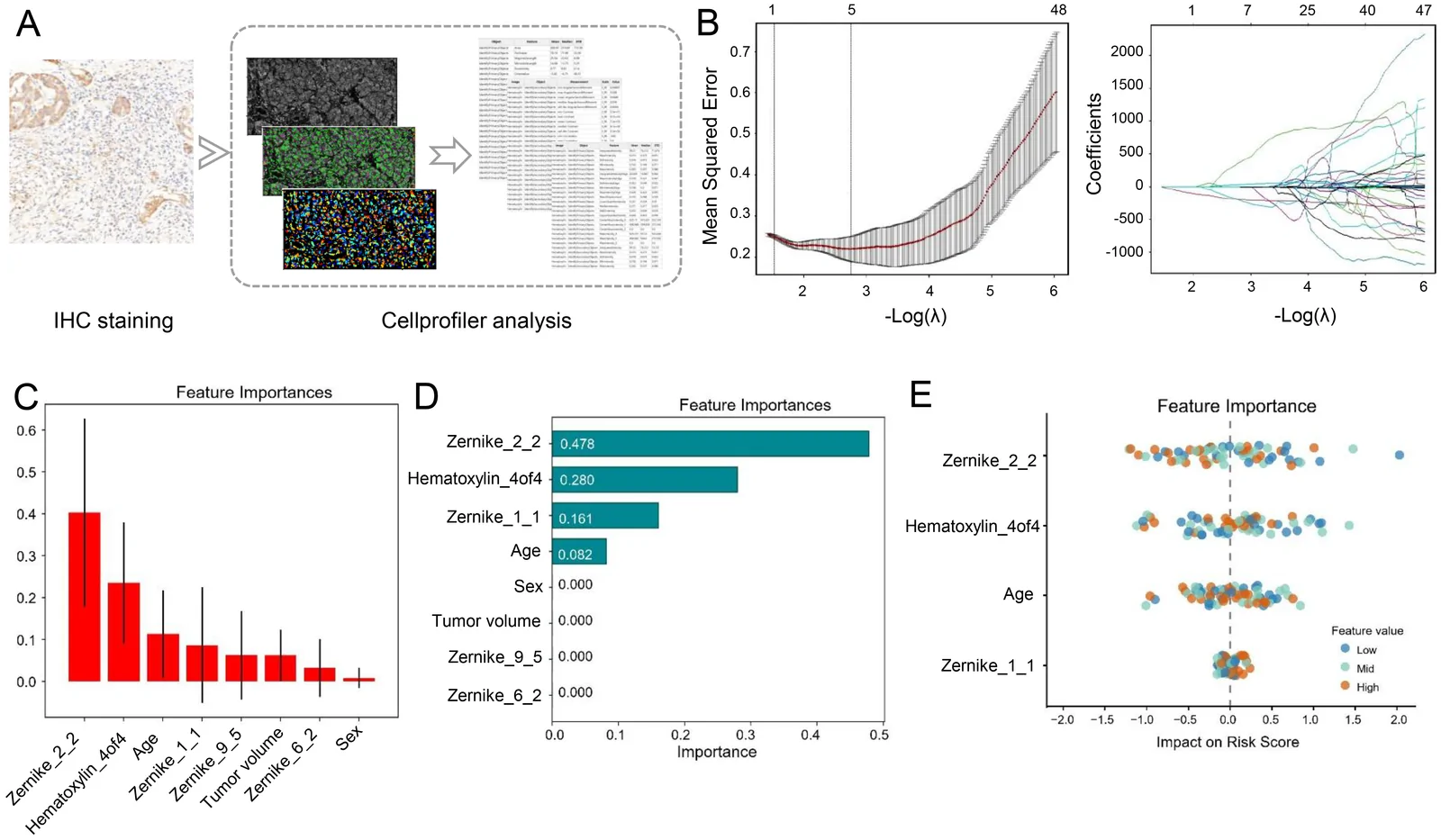
SPP1-associated pathomics feature selection for prognostic modeling. **(A)** Schematic workflow of pathomics feature extraction and model development. IHC-stained tissue sections were processed through CellProfiler for segmentation and feature extraction, followed by ensemble feature selection and model construction. **(B)** LASSO cross-validation curve showing Mean Squared Error plotted against Log(λ). **(C, D)** Feature importance ranking of the eight candidate variables (five pathomics features + three clinical variables) by random forest **(C)** and decision tree **(D)**. **(E)** SHAP analysis showing the contribution of each retained feature in the preliminary four-feature model. SHAP: SHapley Additive exPlanations.

To select the most informative features, we employed an ensemble strategy combining LASSO, random forest, and decision tree (**Figure 3B**). LASSO regression with 10-fold cross-validation (200 λ values, seed = 30) initially identified 5 candidate pathomics features. The feature abbreviations and their full names are listed in **Table 1**. These were then combined with three clinical variables (sex, age, and tumor volume), and random forest and decision tree (both with max_depth = 3 to prevent overfitting) were subsequently applied. Feature importance ranking revealed that Zernike_2_2, Hematoxylin_4of4, Zernike_1_1, and Age were consistently selected by all three algorithms, while Sex, Tumor volume, Zernike_9_5, and Zernike_6_2 showed minimal contributions (**Figures 3C, D**).

**Table 1:** Feature abbreviations and their full names.

Feature full name	Abbreviation
Mean_IdentifyPrimaryObjects_AreaShape_Zernike_2_2	Zernike_2_2
Mean_IdentifyPrimaryObjects_RadialDistribution_RadialCV_Hematoxylin_4of4	Hematoxylin_4of4
Mean_IdentifySecondaryObjects_AreaShape_Zernike_1_1	Zernike_1_1
Mean_IdentifySecondaryObjects_AreaShape_Zernike_6_2	Zernike_6_2
Mean_IdentifySecondaryObjects_AreaShape_Zernike_9_5	Zernike_9_5

A preliminary Cox model was constructed using the four features. Among these, Zernike_1_1 exhibited the weakest contribution (Mean |SHAP| = 0.054) and showed no significant association with overall survival (HR = 0.936, P = 0.749). To enhance model parsimony and mitigate overfitting, this variable was excluded, and the remaining three features—Zernike_2_2, Hematoxylin_4of4, and Age—were retained for final model construction (**Figure 3E**).

### Development and evaluation of the pathomics-based prognostic model

A risk score was derived for each patient as a linear combination of the three selected features weighted by their respective Cox regression coefficients: *Risk Score = (-0.6189) × Zernike_2_2 + (-0.6415) × Hematoxylin_4of4 + 0.0390 × Age*. Based on the median risk score, patients were divided into high-risk (n = 31) and low-risk (n = 32) groups. KM analysis demonstrated a significant survival difference between the two groups (log-rank P < 0.0001; **Figure 4A**), with a C-index of 0.738. The specific coefficients and hazard ratios for each feature are summarized in **Table 2**.

**Table 2:** Multivariate Cox regression analysis of prognostic factors for overall survival.

Variable	Coefficient	SE	HR (95% CI)	P Value
Zernike_2_2	-0.6189	0.1638	0.539 (0.391–0.742)	0.0002
Hematoxylin_4of4	-0.6415	0.2201	0.526 (0.342–0.810)	0.0036
Age	0.0390	0.0193	1.040 (1.001–1.080)	0.0430

Risk Score = (-0.6189) × Zernike_2_2 + (-0.6415) × Hematoxylin_4of4 + 0.0390 × Age.

**Figure 4:**
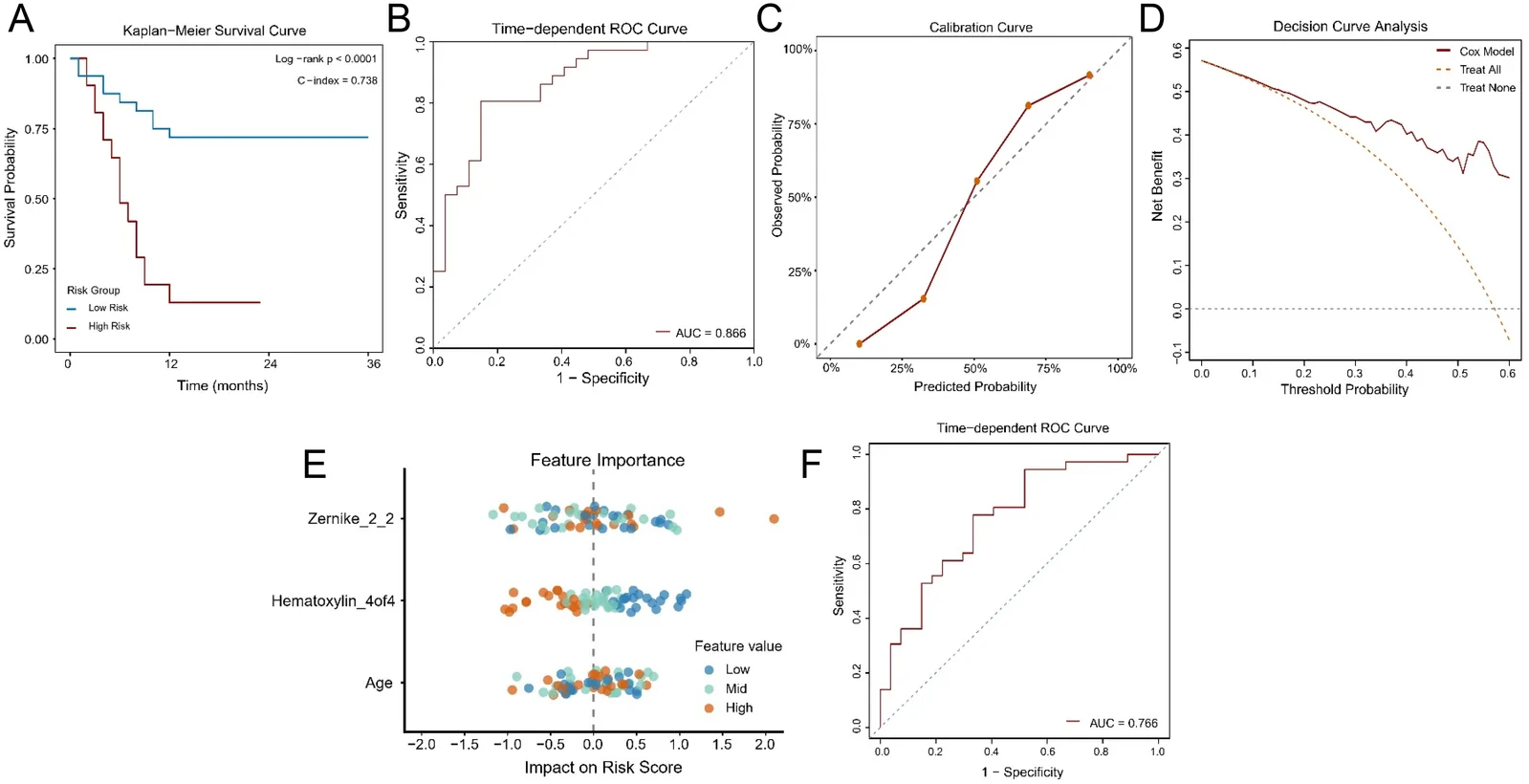
Development and evaluation of the pathomics-based prognostic model. **(A)** KM survival curves for high-risk (n = 31) and low-risk (n = 32) groups stratified by the median risk score of the three-feature model (log-rank P < 0.0001; C-index = 0.738). **(B)** Time-dependent ROC curve at 12 months showing an AUC of 0.866. **(C)** Calibration curve for the model at 12 months. **(D)** DCA demonstrating the net clinical benefit of the model across threshold probabilities. **(E)** SHAP beeswarm plot illustrating the feature importance and directional contribution of each predictor in the final model. **(F)** Bootstrap-corrected time-dependent ROC curve (12 months) with an AUC of 0.766. KM, Kaplan-Meier; ROC, receiver operating characteristic; AUC, area under the curve; DCA, decision curve analysis; SHAP, SHapley Additive exPlanations.

To evaluate model performance, we performed time-dependent ROC analysis, which yielded an AUC of 0.866 at 12 months (**Figure 4B**), indicating good discriminative ability. The calibration curve closely followed the 45-degree reference line (slope = 0.881; **Figure 4C**), suggesting well-calibrated predictions. DCA suggested potential net benefit in this cohort of the model (**Figure 4D**), showing positive net benefits across threshold probabilities from 0 to 0.6 and outperforming both “treat-all” and “treat-none” strategies, particularly between 0.1 and 0.5. To facilitate interpretability, we performed SHAP analysis to evaluate the contribution of each feature. As shown in **Figure 4E**, Zernike_2_2 was the most influential predictor (Mean |SHAP| = 0.475), followed by Hematoxylin_4of4 (Mean |SHAP| = 0.445) and Age (Mean |SHAP| = 0.322). The beeswarm plot further revealed the directional impact of each feature, consistent with the Cox regression results (**Figure 4E**). We next verified the proportional-hazards (PH) assumption for the final Cox model using Schoenfeld residual tests. As shown in **Supplementary Figure S1**, the scaled Schoenfeld residuals for all three variables fluctuated randomly around zero with no apparent trend over time. The global test yielded a P value of 0.055, indicating no strong evidence of PH violation, although this borderline result warrants cautious interpretation.

Finally, we performed internal validation using 1000 bootstrap resampling iterations to correct for optimism. The bias-corrected C-index was 0.715 (95% CI: 0.626–0.801), indicating minimal overfitting. Bootstrap-corrected time-dependent ROC analysis yielded an AUC of 0.766 (95% CI: 0.645–0.877; **Figure 4F**), confirming that the model maintained robust discriminative performance beyond the training cohort.

### SPP1 expression in myeloid cells and macrophage infiltration

To identify cell types expressing SPP1 in PDAC, we analyzed single-cell expression data using the Tumor Immune Single-cell Hub (TISCH2) platform. SPP1 expression was detected across 13 major cell types, with prominent expression in myeloid-lineage immune cells, particularly macrophages and monocytes (**Figure 5A**). This observation was further supported by independent re-analysis of the CRA001160 dataset, which showed elevated SPP1 expression in macrophages and malignant cells (**Figure 5B**). Consistently, visualization via the scCancer Explorer platform indicated predominant SPP1 expression in macrophages and ductal cells (**Figure 5C**).

**Figure 5:**
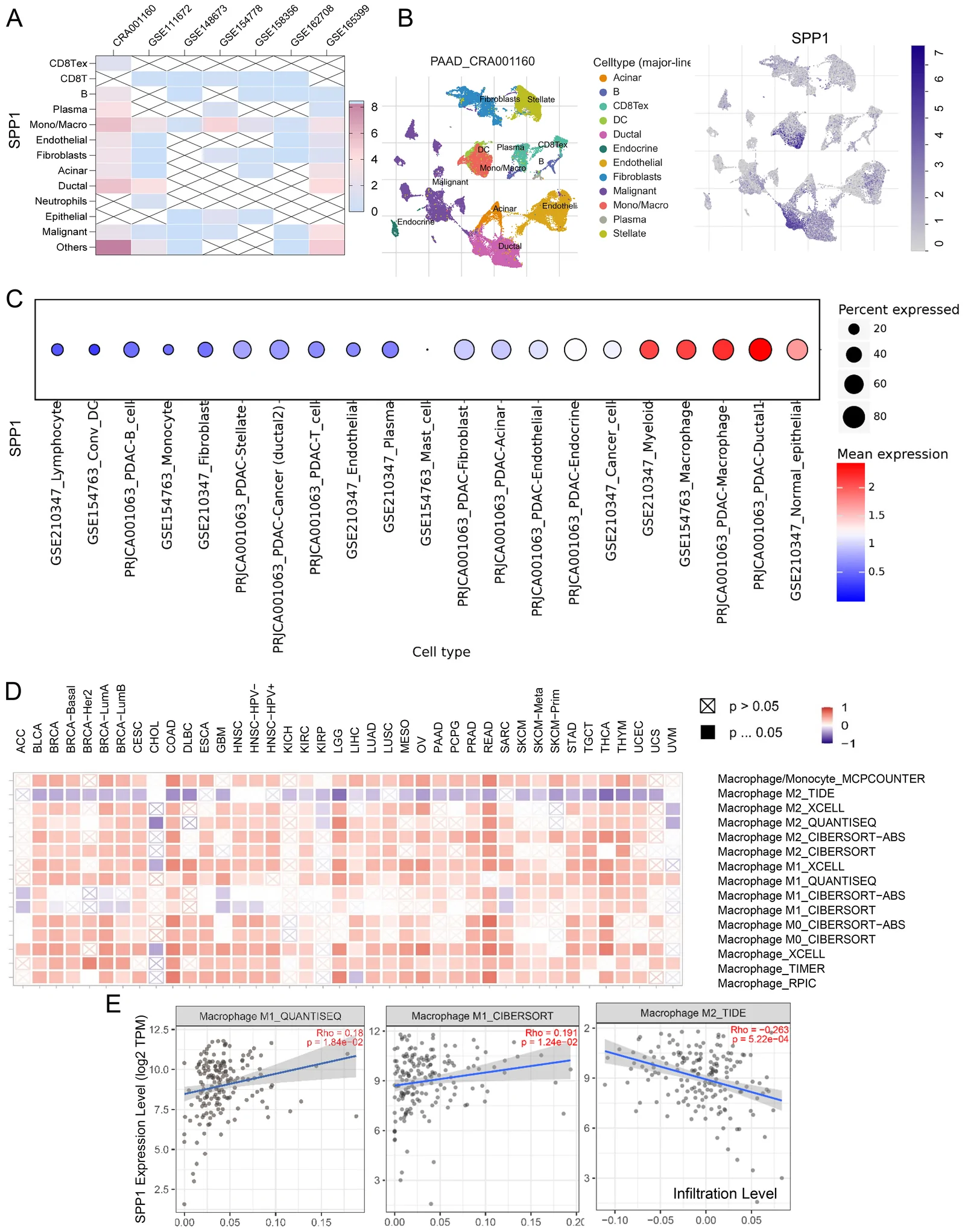
SPP1 expression in myeloid cells and macrophage infiltration. **(A)** Single-cell analysis of SPP1 expression across diverse immune cell populations in PDAC using TISCH2; **(B)** SPP1 expression across cell types in the CRA001160 dataset, showing elevated expression in macrophages and malignant cells, generated using the scCancer Explorer platform; **(C)** Bubble plot illustrating the expression abundance and percentage of SPP1 across various cell types in multiple PDAC datasets, generated using the scCancer Explorer platform; **(D)** Heatmap visualization of SPP1 expression across macrophage subtypes within the TIMER 2.0 framework; **(E)** The infiltration relationships between SPP1 and M1-quanTIseq, M1-CIBERSORT, and M2-TIDE were systematically characterized in TIMER 2.0.

To explore the relationship between SPP1 expression and macrophage polarization in the PDAC TME, we performed immune deconvolution analyses using TIMER2.0, integrating multiple algorithms including quanTIseq, CIBERSORT, and TIDE. Heatmap visualization revealed SPP1 expression across macrophage subtypes in a pan-cancer context (**Figure 5D**). Correlation analyses demonstrated that SPP1 expression was positively associated with both M1 and M2 macrophage infiltration estimates (quanTIseq and CIBERSORT), whereas negative associations were observed with specific M2 subclusters using the TIDE algorithm (**Figure 5E**). These algorithm-dependent associations suggest that SPP1 may be linked to macrophage phenotypic heterogeneity rather than a fixed polarization state.

## Discussion

In this study, we conducted a multidimensional investigation of SPP1 expression in PDAC by integrating public transcriptomic and proteomic datasets, single-cell resources, tissue-based immunohistochemical assessment, and quantitative pathomics analysis. We observed dichotomous expression patterns of SPP1: upregulation in PDAC tumors but downregulation in TLS-enriched regions. More importantly, through a multi-algorithm consensus feature selection strategy, we developed a parsimonious three-feature prognostic model with good discriminative performance and significant survival stratification following internal validation.

Our results revealed that SPP1 expression is lower in TLS-enriched regions compared with adjacent normal tissues. This observation aligns with recent findings identifying SPP1 as a key differentially expressed gene between TLS-high and TLS-low PDAC tumors (11). Since TLSs are organized immune aggregates associated with enhanced anti-tumor immune responses and improved patient outcomes (33), the downregulation of SPP1 within these regions may reflect a local immunomodulatory shift rather than a generalized tumor-intrinsic effect. Notably, SPP1 is not only a TLS-associated gene but also highly expressed in macrophages within the PDAC microenvironment (9, 10), raising the possibility that the observed TLS-related SPP1 reduction could be mediated, at least in part, by macrophage polarization states within TLSs niches. Our immune deconvolution analyses showed that SPP1 expression was associated with macrophage infiltration estimates, consistent with the established role of SPP1 as a potential indicator of tumor immune status. However, the correlation-based analyses do not establish causation, and the observed algorithm-dependent variability, positive correlations with both M1 and M2 estimates in some algorithms versus a negative association with M2 estimates using TIDE, underscores the complexity of macrophage polarization states *in vivo* and cautions against overinterpreting these associations as evidence of fixed polarization. Collectively, these findings suggest that SPP1 reflects the heterogeneous immune landscape of the PDAC TME, encompassing both TLS-associated and myeloid-derived signals, a complexity that motivated our pathomics-based modeling approach.

To operationalize this complexity into a clinically accessible framework, we developed a prognostic model based on handcrafted pathomics features extracted from routine IHC-stained tissue sections. Unlike black-box deep learning architectures that often lack interpretability (20), our model offers greater traceability of feature selection and decision-making, with SHAP analysis clarifying the directional contribution of each predictor, an asset particularly relevant for clinical translation. Using a multi-algorithm consensus strategy that combined LASSO, random forest, and decision tree, we identified three core features (Zernike_2_2, Hematoxylin_4of4, and age) that consistently stratified patients into high- and low-risk groups with significant survival differences. This approach prioritized model parsimony and biological interpretability over purely predictive performance, reflecting our intent to generate hypothesis-driving insights rather than an opaque prognostic score.

As prediction models become increasingly central to clinical decision-making, the methodological framework and reporting strategy of this study align with the growing consensus in the field. TRIPOD provides transparent reporting guidance for developing or validating multivariable prediction models (22), while PROBAST offers structured assessment of risk of bias and applicability, together forming a methodological loop from development to evaluation (23). We followed these frameworks and explicitly identified the lack of external validation as a key limitation, consistent with the exploratory nature of this model-development phase. Similar challenges have been observed in artificial intelligence (AI)-assisted cytological diagnosis of indeterminate thyroid nodules, where insufficient dataset standardization, lack of multicenter validation, and limited algorithm interpretability constrain clinical translation (34). We also acknowledge that our model uses handcrafted pathomics features rather than multiple instance learning (MIL) or foundation models, which can infer immune signals from H&E slides and may improve predictive performance (35). However, such approaches do not always provide explicit biological interpretability. Our model was therefore designed to prioritize interpretability and greater traceability of feature selection and decision-making. Integrating MIL or foundation models with SPP1-associated histoarchitectural analysis, and leveraging digital pathology-based analytics of TME organization (24), represent promising future directions as larger independent cohorts become available.

Several limitations should be acknowledged. First, the single-center retrospective design and relatively small sample size (n = 63) may have limited statistical power, resulting in wide CI for the C-index (95% CI: 0.626–0.801). This underscores the necessity for multicenter external validation in larger, independent cohorts to confirm the model’s generalizability and clinical applicability. Second, the feature set was restricted to three pathomics and clinical variables. Notably, established serum biomarkers such as CA19-9, as well as other potentially important clinical parameters were not incorporated into the model. The inclusion of these variables in future iterations could further improve the model’s predictive accuracy and clinical utility. Third, the absence of spatial transcriptomics or multiplex IHC precludes direct validation of SPP1 localization with TLSs or macrophage subsets in the same tissue sections. Moreover, the immune deconvolution analyses are correlation-based and algorithm-dependent, and therefore do not establish causation. Despite these limitations, we believe this study represents a meaningful exploratory effort to bridge SPP1-associated molecular features with histomorphological patterns through a transparent, interpretable pathomics framework.

In summary, our findings demonstrate that SPP1 is upregulated in PDAC tumors but downregulated in TLS-enriched regions, and is associated with immune features and prognosis. Based on these observations, we integrated SPP1-associated pathomics features through a transparent, multi-algorithm consensus strategy to construct an interpretable prognostic framework that bridges molecular signatures with histomorphology, positioning SPP1 as a promising candidate for further biomarker development in PDAC.

## Data Availability

Publicly available datasets were analyzed in this study. This data can be found here: The publicly available datasets analyzed in this study, including TCGA, GTEx, GEO and CPTAC datasets, are accessible through their respective repositories as cited in the Methods. The pathomics features and clinical data generated during this study can be obtained from the corresponding author upon reasonable request.

## Supplementary material

Supplementary Figure S1 Proportional-hazards assumption test for the final three-feature Cox model. Scaled Schoenfeld residuals for each variable (Age, Hematoxylin_4of4, and Zernike_2_2) plotted against time. The solid red lines represent the smoothed loess curves, with gray shaded areas indicating the 95% CI. The dashed horizontal line at zero indicates no time-dependent effect. A nonzero slope of the smoothed curve would suggest violation of the PH assumption. The global PH test yielded a non-significant result (P = 0.055), indicating that the PH assumption was not violated overall. PH: proportional hazards; CI: confidence interval.
